# Roadmap for an imaging and modelling paediatric study in rural NZ

**DOI:** 10.3389/fphys.2023.1104838

**Published:** 2023-03-10

**Authors:** Haribalan Kumar, Robby Green, Daniel M. Cornfeld, Paul Condron, Taylor Emsden, Ayah Elsayed, Debbie Zhao, Kat Gilbert, Martyn P. Nash, Alys R. Clark, Merryn H. Tawhai, Kelly Burrowes, Rinki Murphy, Maryam Tayebi, Josh McGeown, Eryn Kwon, Vickie Shim, Alan Wang, Julie Choisne, Laura Carman, Thor Besier, Geoffrey Handsfield, Thiranja Prasad Babarenda Gamage, Jiantao Shen, Gonzalo Maso Talou, Soroush Safaei, Jerome J. Maller, Davidson Taylor, Leigh Potter, Samantha J. Holdsworth, Graham A. Wilson

**Affiliations:** ^1^ Mātai Medical Research Institute, Gisborne, New Zealand; ^2^ Auckland Bioengineering Institute, University of Auckland, Auckland, New Zealand; ^3^ GE Healthcare (Australia & New Zealand), Auckland, New Zealand; ^4^ Faculty of Medical and Health Sciences, Centre for Brain Research, University of Auckland, Auckland, New Zealand; ^5^ Auckland University of Technology, Auckland, New Zealand; ^6^ Department of Engineering Science, University of Auckland, Auckland, New Zealand; ^7^ Monash Alfred Psychiatry Research Centre, Melbourne, VIC, Australia

**Keywords:** MRI image analysis, computational modelling, child health and development, neuroimage analysis, radiology

## Abstract

Our study methodology is motivated from three disparate needs: one, imaging studies have existed in silo and study organs but not across organ systems; two, there are gaps in our understanding of paediatric structure and function; three, lack of representative data in New Zealand. Our research aims to address these issues in part, through the combination of magnetic resonance imaging, advanced image processing algorithms and computational modelling. Our study demonstrated the need to take an organ-system approach and scan multiple organs on the same child. We have pilot tested an imaging protocol to be minimally disruptive to the children and demonstrated state-of-the-art image processing and personalized computational models using the imaging data. Our imaging protocol spans brain, lungs, heart, muscle, bones, abdominal and vascular systems. Our initial set of results demonstrated child-specific measurements on one dataset. This work is novel and interesting as we have run multiple computational physiology workflows to generate personalized computational models. Our proposed work is the first step towards achieving the integration of imaging and modelling improving our understanding of the human body in paediatric health and disease.

## 1 Introduction

There are significant gaps in our understanding of paediatric structure and function. Biomedical and health research therefore use or adapt existing non-representative data. This gap in the knowledge base can in part can be addressed through the combination of magnetic resonance imaging (MRI) and computational modelling.

MRI-studies have existed in silo, often only focusing on individual organs. Our study aims to disrupt this trend and take an organ-system approach—by imaging multiple organs on the same subject. Furthermore, paediatric imaging studies are poorly developed in New Zealand, as such the variability in the NZ population (Table 1) has not been adequately described. Furthermore, there is even less data that represents Māori and Pacific children. Hence clinical and health research are left to use or adapt existing non-representative data. Countries such as the Netherlands, Germany, United Kingdom, and United States (Satterthwaite et al., 2014; Sadananthan et al., 2015; Tint et al., 2016; Pausova et al., 2017; Chaarani et al., 2021) are building significant population-based imaging studies, the findings of which will underpin the next-generation of advances in personalized and predictive medicine. These populations will be the first beneficiaries of these advances, and benefit will be greater as findings will be based directly on their population data.

**TABLE 1:** Paediatric imaging studies across the world, their study scope, organs studied and a brief description of the participants.

Study	Design	Imaging	Participants
Generation R Netherlands	Child development study investigating normal and abnormal growth, development and health	Brain, lungs, Abdominal, cardiac	6–11 years old
GUSTO Singapore	Longitudinal studying roles of foetal, developmental and epigenetic factors in pathways to disease	Brain, abdominal; whole body EchoMRI	5-year olds
Saguenay Study Canada	Cross sectional/quasi longitudinal study of adolescents and their parents investigating common cardiometabolic and brain diseases	Brain, abdominal	Adolescents. Participants selected for genetic homogeneity
ABCD Study United States	Understanding development of risk for mental and physical health outcomes	Brain	9–10 year olds. Imaging planned every 2 years

Computational modelling has increasingly been applied to questions of human physiology: modelling has significantly increased our understanding of adult physiology in health and disease, but paediatric applications are very much in their infancy. Computational models are essentially workflows or methodologies that can be employed to derive, infer, or predict structural and/or functional aspects of an organ or organ system. Model-based approaches are powerful tools to understand human structure and function, adding value through *in silico* trials, pre-clinical trials, and help understand disease pathways and biomarkers for their detection, prevention or treatment.

To bridge these two gaps, we first developed an imaging protocol for children. Then state-of-the-art methods were employed to extract measurements from these images. Our protocol spans brain, lungs, heart, muscle, bones, abdominal and vascular systems.

In the longer term, our study will serve to establish normative standards and to improve early diagnosis for paediatric pathologies accounting for an individual’s phenotypic (anatomical and functional) variations. The accompanying data will enable comprehensive tracking and understanding of growth in a young population. Ultimately, our normative imaging and modelling databases can be used to develop predictive and preventative models (Davendralingam et al., 2020; Taylor, 2022) in paediatric medicine.

## 2 Methods

### 2.1 Overall design

**FIGURE 1:**
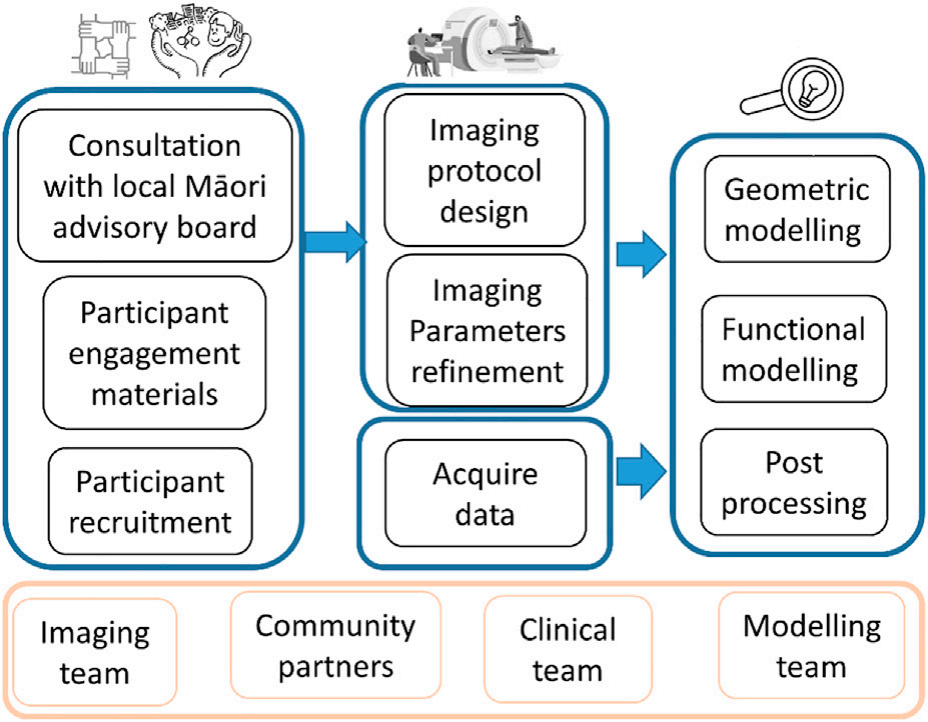
Flowchart showing the overall study design and multiple stages.

This study involved participation from four teams from two institutions including New Zealand community to 1) involve the community and Māori families through co-designing the research protocol and respect Māori culture, 2) finalize and pilot test the imaging protocol to be minimally disruptive to the children, and 3) demonstrate image processing and personalized computational models based on MRI data (Figure 1).

### 2.2 Demographical context

This study was conducted in the Tairāwhiti-Gisborne region located in the eastern coast of New Zealand with an approximate 50% indigenous Māori population living there. It was unclear at the beginning how to implement a population imaging study in the community setting as there was no prior community engagement in the region. Issues of tolerance of scans, cooperation from children and their family were additional complexities.

### 2.3 Participant recruitment

Children resident in the Tairāwhiti-Gisborne District, aged between seven and 12 years old were recruited through convenience sampling. Volunteers with significant congenital abnormalities or morbidity, and inability to tolerate MRI scanning were excluded. Informed consent/assent was gained from parents before participation. Ethical approval was obtained through the Health and Disability Ethic Committee (20/CEN/107). The study was further reviewed by an independent Māori Advisory Board to ensure that the aims and processes are in line with the regional values and priorities. All study scans were reviewed by a consultant radiologist and significant incidental findings were reported to the volunteer’s general practitioner. Participant documentation (consent forms, MRI safety checklist, demographics) were stored on a secure research data management system (REDCap).

### 2.4 Imaging

Images were acquired on a 3T GE SIGNA Premier scanner (General Electric Healthcare, MI, United States), using state-of-the-art 48-channel head coil and Air Coil (AIR™) technologies based at Tairāwhiti-Gisborne, New Zealand. In-bore screen, acoustic shielding, and pre-scan familiarization improved the children’s overall experience.

### 2.5 Imaging protocol

Imaging protocols for each body part were developed in conjunction with the modelling teams. Due to the large number of organ systems of interest and the large number of models being generated, we aimed to incorporate the minimum number of sequences needed for model generation and optimize them for speed and resolution. This was done in an iterative fashion with the modelling teams providing feedback regarding the suitability of images for post-processing as the imaging team made sequence modifications. When the post processing would permit, faster sequences were substituted for slower ones. The final protocol is detailed in Table 2 along with the purpose of each sequence.

**TABLE 2:** Details of image sequences used, with imaging parameters and purpose of the sequence in the study.

Sequence	Scan time	Parameters	Purpose/Link to computational model
**Lung**			
Zero TE (ZTE)	2:00 min	O: Axial	Geometrical model of thoracic cavity, upper airways and prediction of ventilation
*Radial 3D spoiled gradient echo*		TR: 629 ms/TE: 0 ms/FA: 1	
		Res: 1.1 × 1.1 × 1.5 mm	
		Resp triggered	
**Abdomen and Musculoskeletal**			
LAVA FLEX	14 s per station	O: Axial	Bone shape models and extract morphometry
		TR: 4 ms/TE: 1.1 & 2.3 ms/FA: 12°; 1.5 × 1.5 × 1.5 mm	Muscle volume distributions and shape models
*3D spoiled gradient echo with 2 point DIXON*	7 stations head to foot	Breath Hold	Models of fat and iron distribution
		*Autobind - in phase, out of phase, fat and water*	
**Cardiac**			
Short Axis Cine	7 s	O: Short axis stack	Shape and motion models at multiple temporal frames and for biomechanical simulations
(*2D Balanced Steady State Free Precession*)	per slice	TR: 2.8 ms/TE: 1 ms/FA: 50	
		Res: 1.8 × 1.8 × 6 mm skip 4 mm	
	6–10 slices	30 phases/Breath Hold	
Long Axis Cines	7 s per slice	O: Vertical Long Axis	Generating shape and motion models at multiple temporal frames and for biomechanical simulations
[Vertical Long Axis			
4 Chamber		4 Chamber, Left Ventricular Outflow Tract, Right Ventricular Outflow Tract	
3 Chamber		TR: 2.8 ms/TE: 1 ms/FA: 50°	
Right Ventricular Outflow Tract]		ST: 1.8 × 1.8 × 6 mm	
*2D Balanced Steady State Free Precession (bSSFP) cine*		30 phases/Breath Hold	
4D Phase Contrast	6:36 min	O: Axial; TR: 4 ms/TE: 2.2 ms/FA: 8	Non-invasive estimation of cardiac pressures, and validation of computational models
		Res: 1.8 × 1.8 × 1.2 mm overlap 0.8 mm	
		VENC: 160 cm/s; 20 phases; Free Breathing	
**Neuro**			
*3D T2 FLAIR with Real-time Prospective Motion Correction (PROMO)*	2:41 min	O: Sagittal; TR: 6,300 ms/TE: 101 ms	Generating shape model and biomechanical simulations
		TI: 1852 ms/FA: 90	
		Res: 1 × 1 × 0.7 mm	
T1 MPRAGE with PROMO	2:34 min	O: Sagittal; TR: 7.3 ms/TE: 3 ms/FA: 8	Generating shape model and biomechanical simulations
*IR-prep 3D spoiled gradient echo with Prospective Motion Correction*		IR prep time: 1,000 ms	
		Recovery time: 1,012 ms	
		Res: 1 × 1 × 0.8 mm	
2D Diffusion Tensor multi b-value (HARDI post-processing)	3:57 min	O: Axial	Generation of diffusion tracts, and integration with biomechanical simulation models
		TR: 6,248 ms/TE: 70 ms	
		Res: 2 × 2 × 2 mm b = 0, 1,000, 2000, 3000 s/mm^2^ 4, 15,15,30 directions	
3D Inhance Velocity	2:43 min	O: Sagittal; TR: 8.4 ms/TE: 3.6 ms/FA: 10	Generation of vasculature models and centrelines
*3D Phase Contrast*		Res: 0.8 × 0.8 × 1 mm	
		VENC: 40 cm/s	
Amplified MRI (aMRI)	1:31 min	O: Sagittal	Developing understandings of brain movement and stiffness
*3D bSSFP cine*		TR: 2.8 ms/TE: 1.1 ms/FA: 25	
		Res: 1.3 × 1.3 × 1.3 mm	
		20 phases	
2D Phase Contrast	1:03 min	O: Cerebral aqueduct	CSF flow for use in calibrating the brain model + motion
		O:Axial at the level of C2	
4D Phase Contrast	2:00 min	O: Axial; TR: 4.3 ms/TE: 2.4 ms/FA: 8	Cardiac pulsation-induced brain motion (providing blood flow input function)
		Res: 1.2 × 1.2 × 1.2 mm	
		VENC: 80 cm/s; 20 Phases	
Body			
Body coronal T2 (single shot fast spin echo with respiratory triggering through the thorax)	46 per station x 3 stations	O: Coronal; TR: 1,170 ms/TE 90 ms effective/FA: variable Res: 6 × 1.3 × 1.6 mm	General anatomy
Liver IDEAL-IQ			
		O: Axial	
		TR: 4 ms/TE: 1.1 & 2.3 ms/FA: 12°; 1.5 × 1.5 × 1.5 mm	Hepatic iron deposition
		Breath Hold	

FA: Flip angle; O:Orientation; Resp: Respiratory’ TE: Echo time; TR: Repetition time.

Developing research protocols for children can be challenging due to their relatively small size (which inherently limits signal), restlessness, and poor impulse control such as needs to go to the bathroom. Our goal was to implement a comprehensive protocol that could be scanned in two 45-min blocks with minimal breath holds. A short break allows for appropriate coil changes and bathroom breaks. Allowing participants to watch TV (movies, YouTube, Netflix) while inside the scanner was expected to be a major factor in increased compliance.

### 2.6 Feedback to participants

Before scanning, the purpose of the study was explained to the family of the participants. At the end of the imaging session, feedback was collected from participants and their families. Children were shown samples of images acquired during the scans. Accompanying parents and family members were informed about the purpose of the study. A phone call was conducted at the end of the project to update the family on the completion of the pilot.

### 2.7 Image analysis

#### 2.7.1 Brain volume segmentation

For extracting brain volumes, the AccuBrain^®^ software was used. AccuBrain^®^ performs brain structure and tissue segmentation and quantification in a fully automatic mode, has shown better accuracy, efficiency and inter-scanner reproducibility than the widely-used free package FreeSurfer (Abrigo et al., 2019; Liu et al., 2020). T1-MPRAGE and T2 FLAIR with prospective motion correction (PROMO) were adopted for automatic segmentation and quantification of cortical, subcortical, and infratentorial structures based on prior anatomical knowledge specified by experienced radiologists. Absolute volumetric measurements were calculated from the segmentations of the specific brain structures. The relative volumes were calculated as the ratios of the absolute volumetric measurements to the subject’s intracranial volume (ICV) (% of ICV). The high repeatability of this automatic brain structure quantification tool has been validated by brain MR images acquired from different scanners (Zhao et al., 2022). Quantification of myelin was performed using SyMRI (SyntheticMR, Linköping, Sweden) software. R1, R2, and PD maps are generated and input into a four-compartment model (myelin, cellular water, free water, and excess parenchymal water) as in Hagiwara et al. (2017).

#### 2.7.2 Diffusion tractography

Diffusion (dMRI) images were taken through several pre-processing steps using FMRIB’s Diffusion Toolbox (Behrens et al., 2003). Topup (Andersson et al., 2003; Andersson and Sotiropoulos, 2016) was used to estimate susceptibility induced distortion and a distortion field map is estimated. Outputs from Topup and brain mask extracted using BET (Smith, 2002) were fed into the *Eddy* tool (Pajevic and Pierpaoli, 1999) for eddy-current distortion correction. Finally, a diffusion tensor model was created at each voxel by running *DTIFIT*. The distortion-corrected image generated from the previous step was used as an input along with a binary brain mask, bvec (a text file containing gradient diffusion direction information), and bval (a text file containing magnitude of gradient diffusion) files. This tool generated ten files including first three eigenvectors (*ε*_1_, *ε*_2_, *ε*_3_), *λ*_1_ or RD (1st eigenvalue, or radial diffusivity), *λ*_2_ (2nd eigenvalue), *λ*_3_ (3rd eigenvalue), MD (Mean diffusivity), FA (Fractional anisotropy), M0 (mode of anisotropy), and S0 (raw T2 signal). To make the RD, *fslmaths* was applied for averaging L2 and L3 files. FA, MD, AD, and RD maps were used for further analysis. Color maps were generated from the combination of FA map and ε_1_ with conventional color-coding (Smith, 2002). The directionality of fibre tracts is represented in color-coded FA maps by displaying fibres with superior-inferior direction in blue; fibres with anterior-posterior direction in green; and fibres with left-right direction in red.

#### 2.7.3 Brain model methodology

Anatomical T1-weighted scans were used to extract the brain using Brain Extraction Tool (BET) in FSL. Then FMRIB’s Automated Segmentation Tool (Zhang et al., 2001) was used to segment the brain into different tissue types—white matter, grey matter, CSF using a hidden Markov random field model and an associated Expectation-Maximization algorithm (Zhang et al., 2001). The accuracy of this method has been quantitatively evaluated (Kazemi and Noorizadeh, 2014). Diffusion images were then processed with the FMRIB’s Diffusion Toolbox (FDT). They were then processed to extract subject-specific heterogeneous material properties of the brain. The brain tissue was modelled with the hyper-viscoelastic fibre reinforced anisotropic model (Gasser et al., 2006) where the strain energy per unit volume is defined with shear modulus, bulk modulus as well as fibre stiffness. One key feature of this formulation is the fibre dispersion parameter which has been linked with FE measures from diffusion MR images (Giordano and Kleiven, 2014). Using these theoretical frameworks, an automated pipeline that can generate subject-specific FE models of the brain directly from MR images can be employed (Shim et al., 2022). Briefly, our method first customizes a template model of the brain to match the geometry of the subject from the segmented T1-weighted images of the brain using a Free Form Deformation based method. Then each element in the model is automatically assigned with the material property that corresponds the matching MRI voxel in DTI image using the in-house python code, which is based on the automatic material assignment algorithm that we developed for assigning materials to FE models of the bone from CT scans (Shim et al., 2015).

#### 2.7.4 Cerebrovascular and cerebrospinal flow

Delineation of blood vessels and CSF compartments are carried out using CVI42 (Circle Cardiovascular Imaging, AB, Canada) v5.14.1. For blood flow, each image was first preprocessed with a static tissue and mask correction to delineate intracranial blood vessels. Offset correction and anti-aliasing were also applied to reduce image degradation from phase offsets and aliasing artefacts. Each blood vessel was segmented manually by tracing the vessel at a start point and with subsequent points until the end point of the vessel was reached. Each point was adjusted in the sagittal, axial, and coronal plane to ensure it was in the center of the vessel lumen. Blood flow analysis was performed by manually placing regions of interest (ROI) throughout the length of each segmented vessel. ROIs were positioned perpendicular to the presumed direction of blood flow. An automatic contour of the vessel cross-section from the ROI placement was generated for flow calculation in each cardiac phase. Measurements of blood flow, velocity, and kinetic energy of flow were automatically calculated for each ROI throughout the 20 cardiac phases.

For 2D flow, a ROI was manually traced around the CSF compartments of the aqueduct and the subarachnoid space at the level of C2 on a single slice. A static tissue mask correction was applied to better delineate the CSF compartments and aid in tracing of the compartment contour. Background correction and anti-aliasing correction was also applied to reduce inherent errors during acquisition such as eddy currents, gradient field effects and phase offset as well as reducing aliasing artefacts that can decrease the accuracy of flow parameters. The ROI contour was forwarded into the 30 cardiac phases and measurements of CSF flow and velocity were automatically generated throughout the cardiac phases.

#### 2.7.5 Extracting brain motion

Brain motion was extracted from the aMRI sequence using two steps. Cine images are amplified by employing a previously published (Abderezaei et al., 2021; Terem et al., 2021) phase-based video processing method. An amplification factor of 30 was used. This amplification factor supports sufficient amplification with minimum artefacts and distortions. Dynamic displacement field of the amplified brain motion was calculated using intensity-based image registration. Each cardiac frame was registered to the first cardiac frame resulting in a Lagrangian displacement field.

#### 2.7.6 Vascular model methodology

Arterial and venous systems were manually segmented from 3D Phase Contrast sequence using 3D Slicer. The centerlines were then extracted using SlicerVMTK extension. Radius and length for each vessel were automatically computed using an in-house Python script from the smoothed segmentation of the vasculature for each subject.

#### 2.7.7 Cardiac modelling methodology

Child-specific biventricular mathematical models were generated from short and long axis cardiac MR images using a semi-automated segmentation workflow in Cardiac Image Modeler (CIM) (v8.3.0, University of Auckland, New Zealand) (Young et al., 2000). User identified anatomical landmarks were located and automatically tracked throughout the cardiac cycle using non-rigid registration. These landmarks included the four valves, the epicardial apex, right ventricular inserts, and center points. Guide-points were then placed to deform the automatically fitted model to match those points in real-time to end diastolic and end systolic volumes. RV and LV endocardial and epicardial surfaces were fitted, while the papillary muscles were excluded from the myocardial mass. Cardiac indices, such as end diastolic, end systolic and stroke volumes, as well as ventricular mass were calculated using numerical integration (Dyverfeldt et al., 2015; Mauger et al., 2019; Mauger et al., 2021; Elsayed et al., 2022).

Flow acquisitions were processed using 4D Flow Demonstrator V2.3 software (Siemens AG, Erlangen, Germany). Corrections used were antialiasing and background phase correction providing a range of flow measurements of up to 320 cm/s without aliasing effects. Forward and reverse volumes across the pulmonary and aortic valves were quantified. Velocities across the valves were also measured (Dyverfeldt et al., 2015).

#### 2.7.8 Lung modelling methodology

Lung and airways were segmented using Pulmonary ToolKit (PTK, available at: https://github.com/tomdoel/pulmonarytoolkit.git). Further, the main bronchi are identified during segmentation, thereby separating the left and right lung. Next, a 3D surface geometrical model of the lung is chosen as a template mesh with predefined topology. The left and right lung segmentations are fitted to this template geometry following a method previously described (Minaeizaeim et al., 2019). Next, the trachea and upper airways (to the first sub-segmental bronchi) were represented using a predefined template, i.e., a connected set of lines consisting of 69 nodes and 68 elements placed at the centerlines of the upper airway tree. An upper pulmonary arterial tree was derived to match the upper airway template. Finally, the subject-specific lung fitted lung shape and subject-based upper airway and arterial trees were used to generate anatomically consistent airway and arterial trees—from the trachea or main pulmonary artery to the terminal conducting bronchioles and their accompanying arterioles—using a previously published volume-filling algorithm (Tawhai et al., 2004). Tissue perfusion was simulated using the arterial tree geometry and a published model for simulating pulmonary blood flow (Clark et al., 2011; Clark et al., 2019). The geometric and perfusion model methods use the same approaches as have previously been used to model adult lung anatomy. For demonstration purposes, a 15 mmHg mean arterial inlet pressure, 6 mmHg venous pressure and a venous side outlet radius of 12 mm was assumed. Blood flow in the ladder-model was simulated in the supine posture.

#### 2.7.9 Bone modelling methodology

Pelvis, femurs, tibias and fibulas were manually segmented from the LAVA Flex Autobind WATER sequence using Stradview (University of Cambridge, United Kingdom) (Cignoni et al., 2008). The resulting point cloud was smoothed and remeshed using Meshlab (Carman et al., 2022). Each of the bones meshes in the dataset were fitted to a template mesh (one for the pelvis, one for the femur and one for the tibia/fibula) to achieve nodal correspondence for each bone. The template mesh is chosen as the mesh with the desired number of nodes and node distributions for which all meshes in the dataset will be fitted to. This was achieved by non-rigidly registering the template model and iteratively fitting the template mesh to the segmented data using radial basis functions (Carman et al., 2022). Next, all bones were rigidly aligned in accordance with their individual center of mass to remove rotational and translational variations. The final step was to perform a PCA on the aligned meshes to generate the mean mesh and the principal components of variation in the dataset.

In order to understand morphological changes in bone shape and clinical measurements in the dataset, each bone (pelvis, femur and tibia/fibula) was aligned according to their International Society of Biomechanics coordinate systems convention (Wu et al., 2002) using automatic detection of bony landmarks calculated from each 3D mesh to ensure all measures were in the same orientation. Bone measurements were automatically computed using an in-house Python code from the calculated 3D landmarks for each mesh, as described below:

Angular and torsional measurements.

• Anteversion angle: angle between the neck axis of the femur (measured between a sphere fit to the femoral head and the center of a cylinder fitted to the femoral neck), and the posterior condylar axis (measured between the medial and lateral posterior femoral condyle.
• Neck shaft angle: angle between the neck axis of the femur and the shaft axis of the femur (measured between the center of a cylinder fit to the femoral shaft below the lower trochanter and the midpoint of the center of a cylinder fit to each femoral condyle).
• Femoral mechanical angle: angle between the knee axis of the femur (measured between the most distal points on the medial and lateral femoral condyles) and the *y*-axis (measured between the femoral head center and the condylar midpoint).
• Tibial torsion: angle between the posterior condylar axis (measured between the medial and lateral posterior tibial condyle) and the malleolar axis (measured between the medial and lateral malleolus).
• Tibial mechanical angle: angle between the knee axis of the tibia (measured between the centres of a cylinder fit to the medial and lateral tibial condyle) and the *y*-axis (measured between the midpoint of a cylinder fit to the medial and lateral tibial condyle and the midpoint between the medial and lateral malleolus).

Additionally, linear measurements included Anterior Superior Iliac Spine (ASIS) width, Posterior Superior Iliac Spine (PSIS) width, pelvis depth, hip joint center distance, femoral head diameter, femoral length, epicondylar width, condylar width, tibial length, and malleolar width.

#### 2.7.10 Muscle models methodology

To understand paediatric development, growth, and overall physical fitness, skeletal muscle volumes were determined and evaluated from MRI data. We segmented individual muscles in MR images acquired using the LAVA Flex Autobind WATER sequence. Segmentation was performed semi-automatically using 3D Slicer (Fedorov et al., 2012) by manually identifying muscle boundaries in axial slices at several locations through the muscle length. The interpolation function in 3D Slicer was then used to segment muscles between manually segmented slices. All slices were visually inspected by a trained user to ensure accuracy and the 3D rendered muscles were further inspected for any apparent errors. After generation of 3D muscle models, muscle volume data can be used to determine size scaling relationships, normative muscle volume profiles, abnormality for pathological populations, assessment of activity-based strength profiles, or statistical shape modeling.

## 3 Results

**FIGURE 2:**
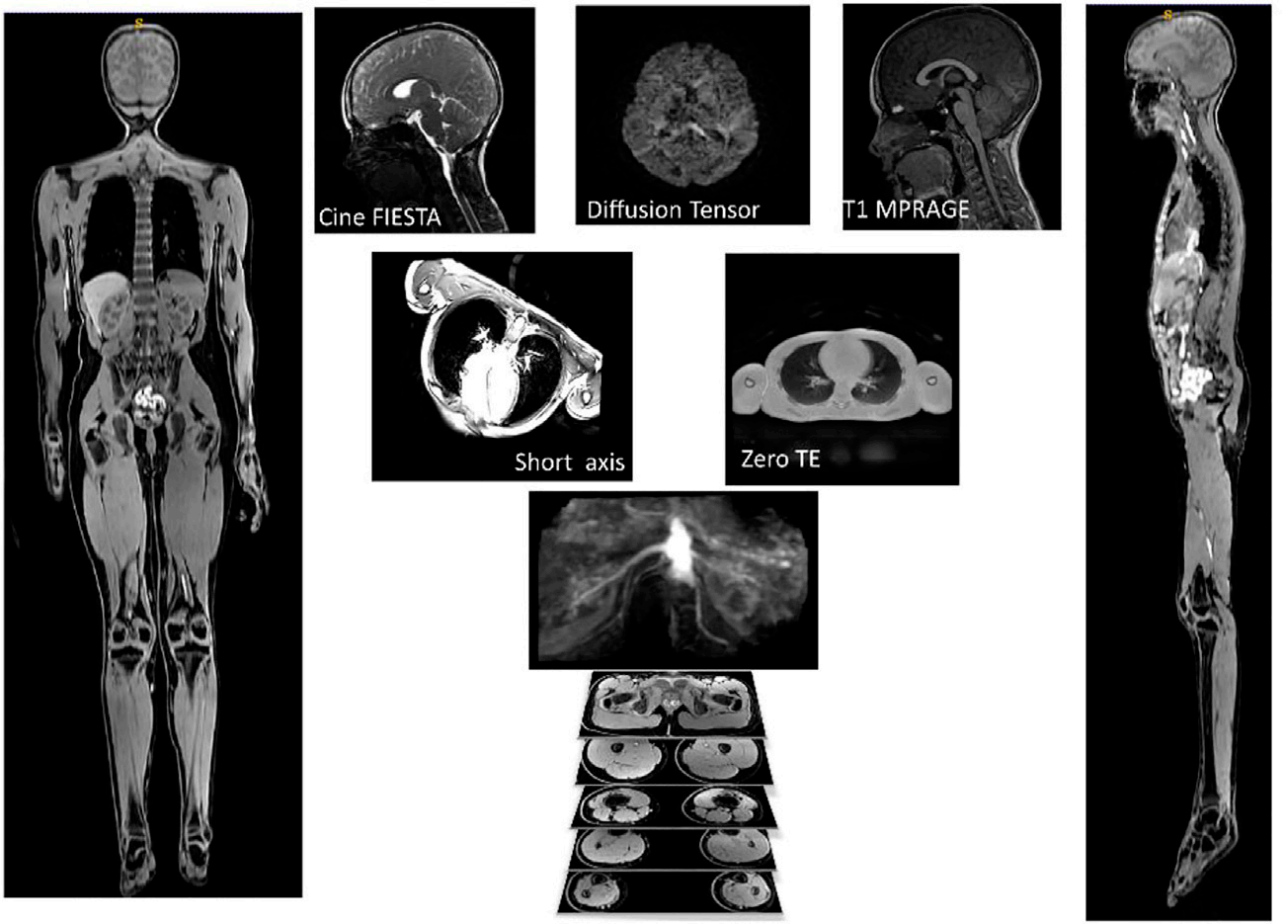
Example of images used for one participant. AutoBind whole body images on the right and left was used for bones and muscle, top three pictures (Cine FIESTA, DTI and T1 MPRAGE) were used to model the brain, under the brain are two pictures of the sequences (Short Axis and Zero TE) used for the heart and lungs.

The final image protocol is shown in Table 2. From the 31 children participants recruited, one child could not tolerate the MRI scan, finding the breath holds particularly uncomfortable. Several children struggled with the breath holds during cardiac and lung imaging. Children and their parents reported positively on the ability to watch video during scan, having break during protocol with juice and snack and showing children samples of images acquired during the scans. Sample images from the sequences are shown in Figure 2.

### 3.1 Exemplar subcortical volume result

Table 3 shows the relative values of various regions of interest. The relative volumes were obtained through calculating the ratio of the absolute volume to intracranial volume (ICV). The left and right relative measurements were generated for comparison of the asymmetry between the left and right hemispheres. The total relative measurements were obtained by the sum of the left and right relative volumetric measurements.

**TABLE 3:** Relative and absolute volume measurements from Accubrain.

	Relative (Left, Right)		Relative total
Cingulate	(1.22, 1.36)	Pons	0.903
Frontal	(8.35, 8.24)	Hippocampus	0.4
Insular	(0.66, 0.625)	Cerebellum	10.4
Occipetal	(3.92, 3.8)	Parenchyma	92.25
Parietal	(0.122, 0.118)	CSF	20.5
Pallidum	(0.173, 0.168)	WM	41.9
Putamen	(0.436, 0.47)	GM	48.3
Caudate	(0.221, 0.21)		
Amygdala	(0.124, 0.124)		
Thalamus	(0.41, 0.354)		

### 3.2 Sample tractography results

Average diffusion tractography values of the corpus callosum are shown in Table 4. These include the fractional anisotropy (FA), mean diffusivity (MD), axial diffusivity (AD), and radial diffusivity (RD). Least amount of variation across the corpus callosum is the mean RD with a standard deviation percentage variation of about 35%. AD showed the highest amount of variation with a percentage variation of 169%. This is followed by MD which showed a 143% variation and FA which had a percentage variation of about 58%. Our results showed that FA had a very low amount of variation compared to AD and MD. This is an important finding as it suggests that FA, which is by far the most widely used DTI metric, may not be the most relevant when investigating white matter based on diffusion imaging. This is consistent with others (Maller et al., 2014; Sarica et al., 2019) who reported a similar conclusion.

**TABLE 4:** Tractography values for corpus callossum fibers.

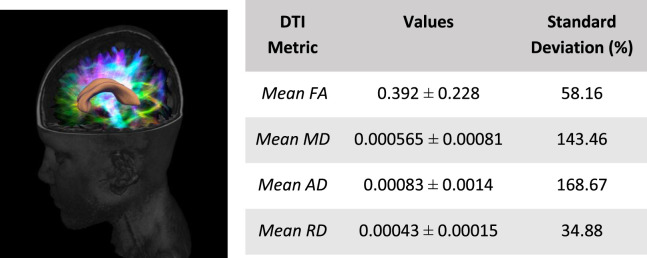

### 3.3 Child-specific brain model results

**FIGURE 3:**
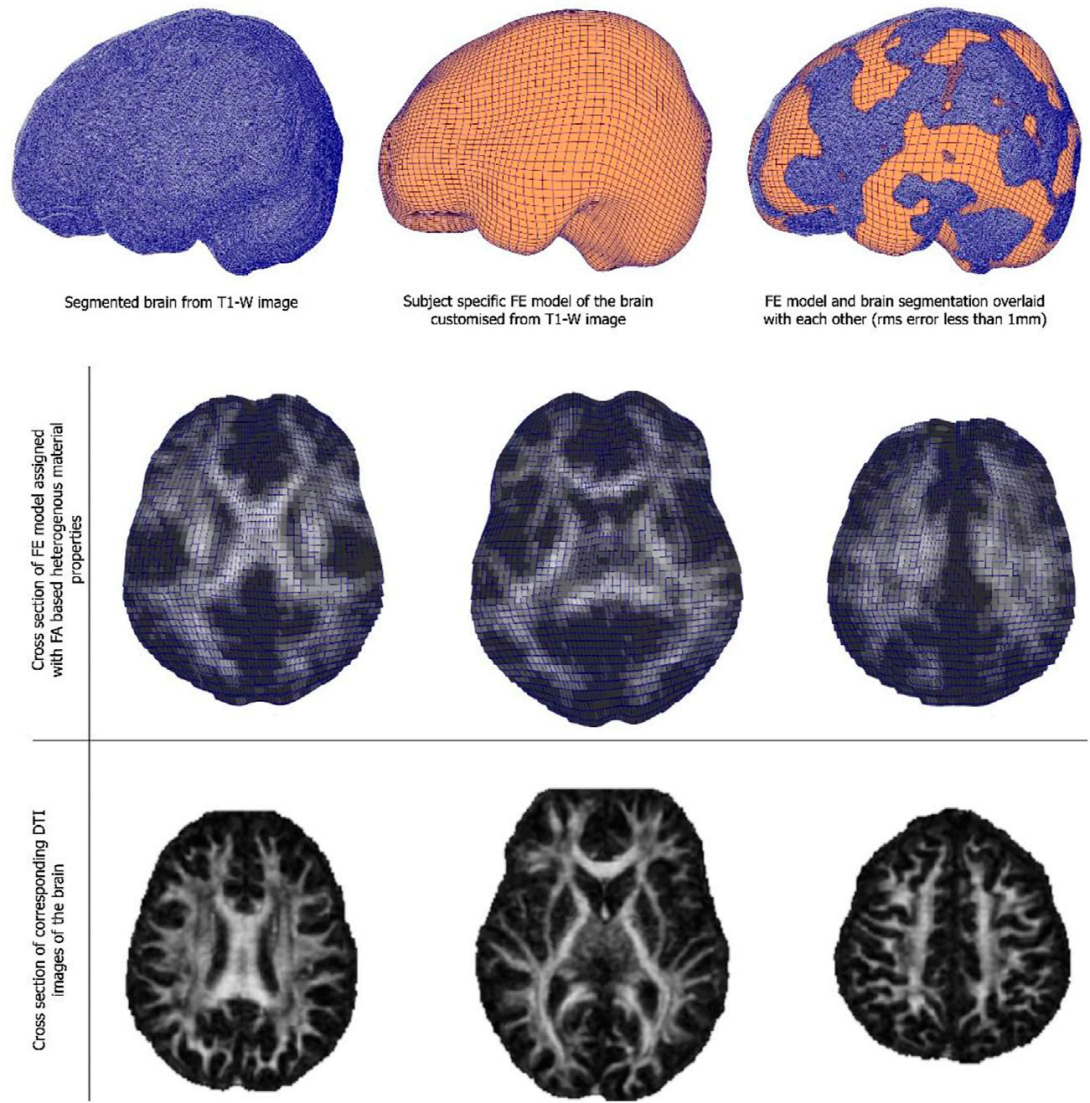
(Top and middle row) An exemplar case of child-specific brain model customized to an anatomical T1-weighted image and FE model overlaid. (Bottom row) Cross section of the diffusion tractography (DTI) images with FA-based heterogeneous material properties.

Customised brain model results are shown in Figure 3. The model customization to the segmented brain image was performed with the accuracy of less than 1 mm RMS error between the model and the segmented images. Figure 3 shows the material property assignment using the FA values of the DTI images. It demonstrates how the model captures subject-specific heterogeneous anisotropy of the brain.

### 3.4 Dynamics-brain motion, cerebrovascular and cerebrospinal flow profiles

**FIGURE 4:**
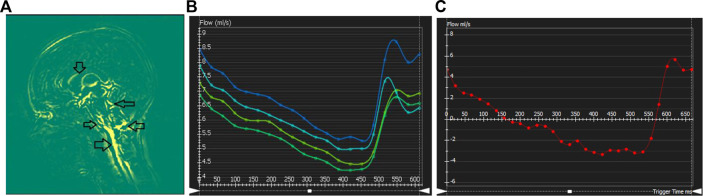
Cerebral dynamics measured from cardiac-gated sequences: **(A)** Mid-sagittal slice showing maximum difference map depicting mid-brain motion. Arrows showing motion in the Pons-CSF interface, Medula Oblongata CSF interface, brain stem motion, motion in the cerebellar tonsil and lateral ventricle CSF boundary. **(B–C)** Flow processing for one representative subject. Plots show blood flow over the cardiac cycle in the basilar artery **(B)**, as well as CSF flow **(C)** over the cardiac cycle at the level of C2.

Brain motion, blood flow profile, CSF flow profile measured through the C2 cervical level are shown in Figure 4. Negative CSF flow values show caudocranial flow direction and positive values show cranio-caudal flow direction. Flow through the left and right ICA and basilar artery represents arterial blood flow. The temporal pattern of the flow curves are similar with a systolic peak and a diastolic peak.

### 3.5 Cardiac analysis results

**FIGURE 5:**
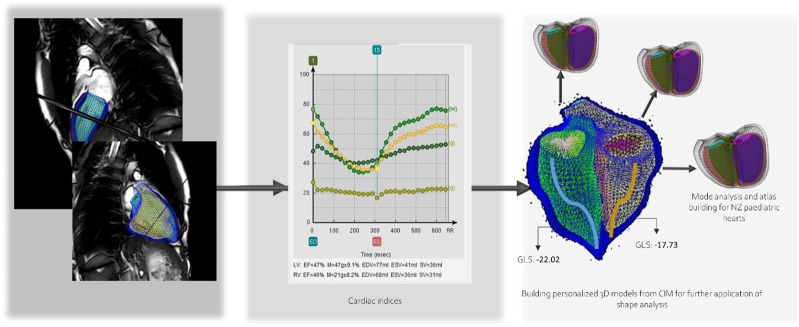
Child-specific cardiac ventricular model and extracted indices.

Cardiac analysis included cardiac indices such as end diastolic volume, end systolic volume, stroke volume, ventricular mass, and ejection fraction, and the building of the 3D personalized model of each subject. Global longitudinal strain was calculated from the model. Then flow measurements such as forward and backward volumes and velocities were calculated for the aorta, pulmonary artery and branches, pulmonary veins, and the vena cava. An analysis of valvular flow of the mitral and tricuspid was also performed. 4D flow data was then combined with the cardiac model to further test the new indices that could be calculated for flow and energy in the ventricles. Figure 5 shows the indices and the cardiac model workflow.

### 3.6 Vascular results

Table 5 shows the radii and lengths measurements of cerebral vasculature (Circle of Willis). The measurements for some arteries are missing due to low resolution of such small vessels in the MR images. Note that further manual curation might be needed to identify if the participant has a missing vessel.

**TABLE 5:** (Top row) Radius measurements of cerebral vasculature in circle of willis region (CoW). The measurements are reported in centimeters. (Bottom row) Lengths measurements of cerebral vasculature in CoW. The measurements are reported in centimeters.

	Left ICA	Right ICA	Left MCA1		Right MCA1	Left ACA1		Right ACA1	Left ACA2		Right ACA2		BA		Left PCA1		Right PCA1		Left PCA2	Right PCA2	Left PcoA	Right PcoA
	2.81	2.72	1.86		1.86	2.01		1.64	2.46		2.46		1.92		1.47		1.31		—	1.52	—	1.44

Left MCA1		Right MCA1		Left ACA1	Right ACA1	Left ACA2		Right ACA2		BA		Left PCA1		Right PCA1		Left PCA2		Right PCA2		Left PcoA	Right PcoA	
4.88		7.81		18.80	18.26		4.01	4.01		32.66		27.49		12.92		—		19.72		—	11.39	

ICA, internal carotid artery; MCA, middle cerebral artery; ACA, anterior cerebral artery; PCA, posterior communicating artery; BA, basilar artery; PcoA, posterior communicating artery.

### 3.7 Lung model results

**FIGURE 6:**
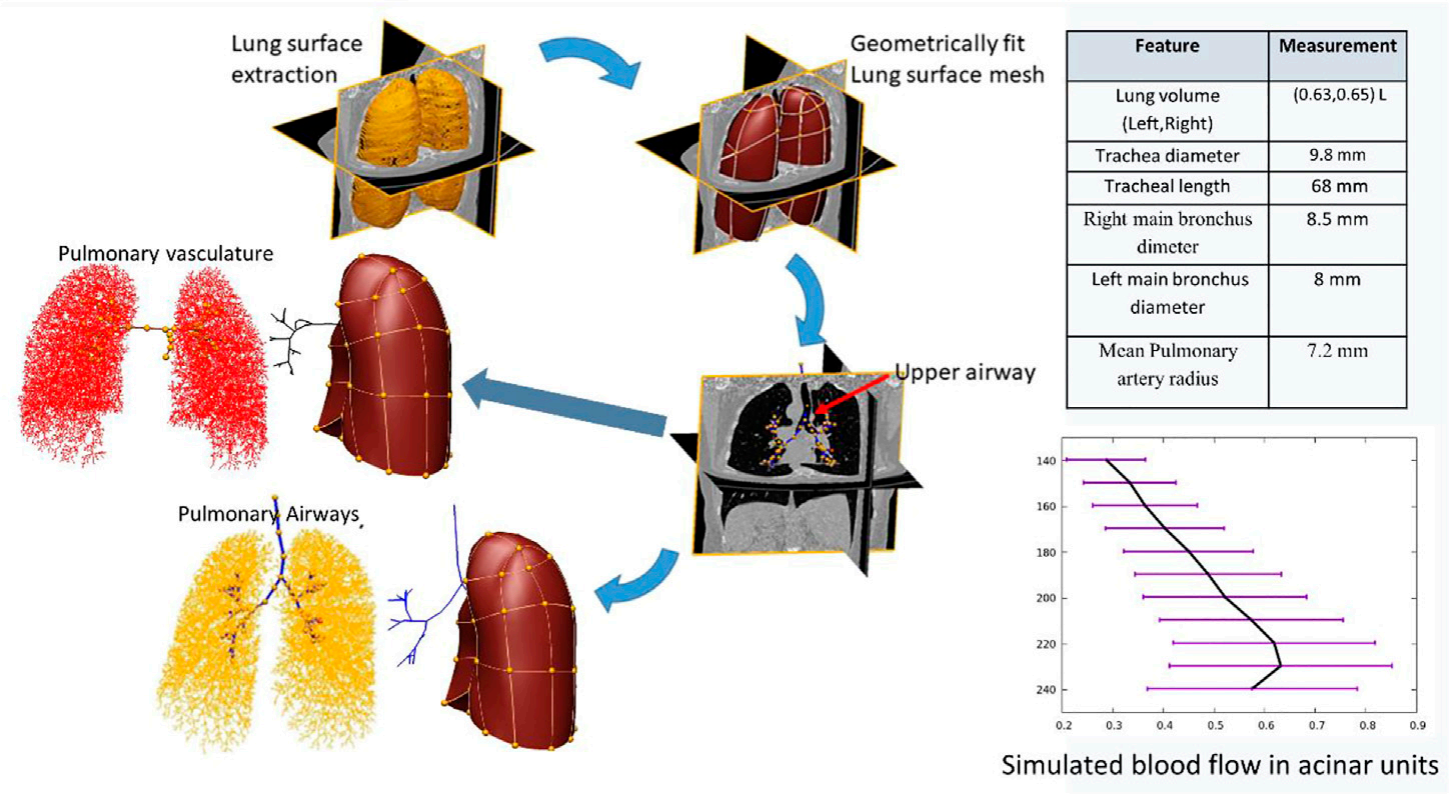
Flow chart showing the generation of child-specific airway and vascular geometries.

Figure 6 shows a child-specific 3D lung geometry, airway, and vascular tree. The airway and vascular tree each consists of a total of about 60,000 segments covering the entire thoracic cavity. Extracted measurements from the model and simulated blood flow in the terminal units are also plotted. The mean and standard deviation of blood flow calculated within 10-mm iso-gravitational or anterior-posterior direction is shown. The model predicts a heterogeneous perfusion distribution within iso-gravitational slices as illustrated by the error bars (±SD) with increasing blood flow down most of the gravitational height of the lung, and a section of decreasing flow in the most gravitationally dependent lung region.

### 3.8 Bone model results

**FIGURE 7:**
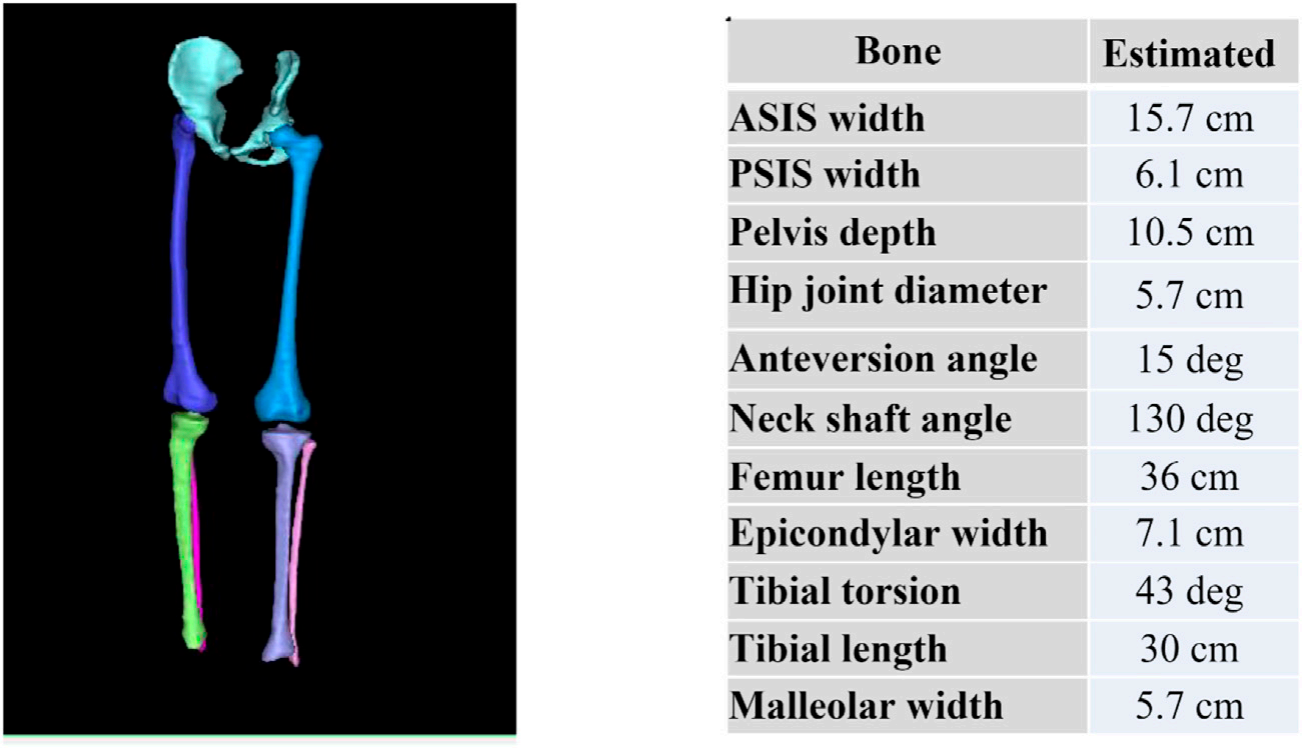
Reconstructed pelvis, femurs, tibias and fibulas from MRI and subsequently fitted with template meshes (left) to automatically compute bone torsional and linear measurements (right).

Resulting mesh fitted bones for one participant is shown in Figure 7. From these mesh fitted bones, we can automatically extract and display bones’ angular and torsional measurements as shown on Figure 7. Understanding angular and torsional measurement in the typically developed skeleton is important to define what is considered “normal.” We aim to use this dataset across ethnicity, age, and gender to build an atlas of “normal” range for these values to empower clinicians in their understanding of the ‘normal range’ values across ethnicity, gender, and age.

### 3.9 Muscle model results

**FIGURE 8:**
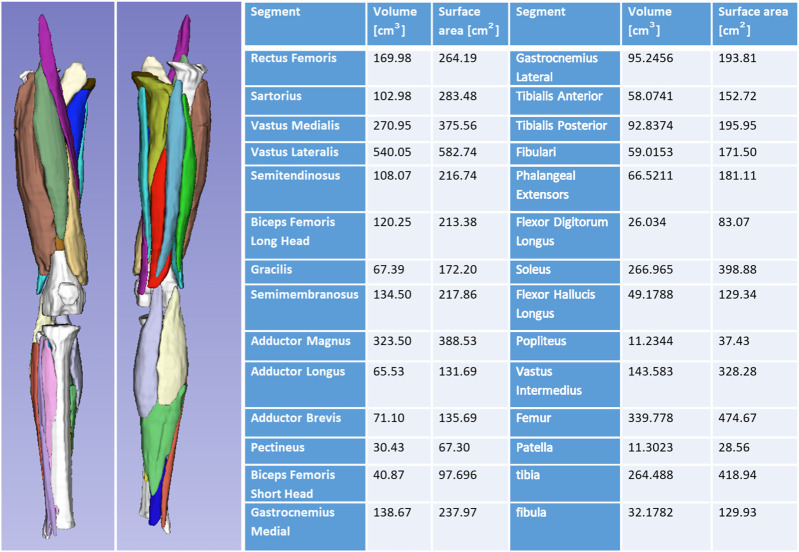
3D volume rendering of the lower limb skeletal muscles and bones offers insight into the musculoskeletal structure of the patient. Volumes can be analyzed and compared against normative datasets to assess muscle size abnormality. 3D surface data offers information on muscle shape and can be used for statistical shape modeling.

Figure 8 shows generated 3D muscle models from MRI in the lower limb muscles. Twenty-five muscles and three bones were successfully rendered from T1-LAVA data from a 3T MRI scanner. Muscles rendered in 3D demonstrate the overall geometry, size, and shape of the lower limb muscles (Figure 8). Segmentation of individual muscles is a time-intensive process that generally involves manual user input. In carrying out this segmentation, data from individual muscles are available offering the ability to assess individual muscle size against normative databases to determine individual muscle abnormalities for the patient. Shape data generated from individual muscle segmentation may be used to conduct statistical shape modeling of individual muscles. Muscle data also provides opportunities for correlating muscle size with other organ physiology in longitudinal studies, offering insight into the ways that skeletal muscles grow with other tissues and organs in the body.

## 4 Discussion

This pilot study demonstrated that a child scanning initiative can be implemented in a community setting. This prospective pilot study demonstrated the first paediatric models in a representative population (Māori and New Zealand European) from MRI imaging that can mimic both structure and function in a robust yet physiologically accurate manner. This study is novel because it has not been undertaken in NZ before; incorporated state-of-the-art MR imaging sequences; examined multiple organ systems (head to toe) within the same scan session; showed single-session multiple organ imaging to be possible in children as young as seven. In some organs, it showcased use of a “model-based” approach to derive structural or functional information that otherwise would not be possible just from images. In other cases, it extracted useful information using advanced post-processing methods.

Imaging challenges: Imaging children with MRI comes with its own challenges. For example, the imaging of lung tissues with ZTE (Hatabu et al., 2020) which is an ultrashort T2/T2*breathing is a relatively new technique but holds huge promise for lung imaging. Lung MRI (Dournes et al., 2021) can be used clinically to assess cystic fibrosis also allowing for quantification of disease severity. Hence use of lung MRI in combination with advanced structural and functional models is justified. Cardiac MRI sessions are typically time-consuming to acquire, with many exams taking over an hour. Participants need to lie still for the duration of the scan, and involves breath holds which makes it difficult for participants who are fidgety or fearful especially for children. Our imaging protocol has been refined with this aspect in mind. Newer image reconstruction techniques such as using deep learning can reduce the number of breath-holds required to obtain the images needed for model generation from approximately 20 breath-holds to five; with some of the breath holds only lasting 2–4 s. Images using these newer techniques were of acceptable quality for post-processing, model generation and extraction of measurements.

Impacts: The long-term aim of this study is to develop an imaging protocol and knowledge database by combining state-of-the-art imaging tools with cutting edge modelling. Our research process will shift knowledge from reactive approaches to more personalized and preventative one that relies on data derived from a rural New Zealand population. Additionally, we believe this project will have an impact on health education and benefit participants who, through their engagement with researchers, will understand the value of their participation and provide feedback.

### 4.1 Impact of brain physiology and structure measurement and models

Brain MRI is used in clinical research studies to examine a vast range of neurological conditions, including understanding common brain diseases, neurodevelopment, illness progression, and the effect of medical interventions. However, physiological factors such as hydration and time of day may cause small changes in brain volume, function, and hemodynamics. Providing a normative database of structural and physiological metrics can thereby provide a benchmark against which phenotypical differences can be elucidated and increase the ability of imaging data to provide evidence of beneficial and adverse outcomes of interventions or treatments. However, there is a lack of such normative databases, in particular for paediatric populations.

Our study demonstrated that it is feasible to include a range of neuroimaging sequences in paediatrics that probe brain structure, microstructure, motion, and physiology. Such a wide and complementary set of neuroimaging methods will enable understanding of the impact of childhood environments on brain development.

Our study is the first of its kind to apply 4D blood flow and CSF flow imaging along with amplified MRI (to visualize brain tissue motion) in children. All three methods were acquired using a cine method to capture the dynamics of these three key compartments that impact brain mechanics. Understanding normative variation of blood flow, CSF flow, and tissue motion are thought to be important for the understanding and diagnosis of several diseases, such as those that cause an elevation in intracranial pressure. Understanding the interplay between the physiology of the brain and its intrinsic motion is thought to assist with understanding various brain pathologies such as in obstructive brain disorders, and diseases which may alter the intracranial pressure (ICP). Changes in ICP are attributed to volume changes in one or more of the constituent tissues contained in the cranium. Causes of increased ICP include a space occupying lesion (e.g., brain tumour); generalized brain swelling (e.g., hypertensive encephalopathy and brain trauma); obstruction to cerebrospinal fluid (CSF) flow (e.g., Chiari malformation or meningitis) and idiopathic intracranial hypertension (IIH). Providing normative metrics of physiological factors that might vary ICP across children will assist in modelling the dynamics of brain tissue, blood flow, and CSF and their influence on ICP.

Diffusion tensor imaging (DTI) has revolutionized our understanding of white matter anatomy, structure-function associations in the nervous system, neurological disease, and developmental problems after pre-term delivery. More recently myelin MRI technology has been developed to study alterations in myelin as occurs in maturation and disease states (Warntjes et al., 2016). DTI and myelin studies are refining the way white matter development is understood. It is generally thought that myelination limits developmental change and plasticity in axons. Therefore, delayed myelination as tracked by such techniques, may help to explain the continued growth of executive abilities (such as planning, organizing, inhibiting automatic response) into adulthood. Indeed, DTI can be used to link features of white matter maturation to cognitive function.

Despite these discoveries and advances in the way we can image and track white matter development with MRI, further research is needed before DTI can be used routinely for clinical purposes. At the present time, DTI is considered prone to variation across scanners, different centers and scanning and image-processing protocols which has hindered the application of DTI in the clinics. DTI has also not been applied widely in diverse populations. Building a normative database using one scanner and across a diverse population could help to mitigate such differences and help to explain the variation in disparate findings.

The axonal structural anisotropy represented in DTI indices can also provide deeper insight into the mechanical responses of the brain after traumatic brain injury (TBI). The strong anisotropy observed in white matter fibre tracts has been shown to influence strain distributions of the brain after head trauma. This led to the development of a computational framework that can incorporate FA values from the DT MR images to achieve heterogeneous and anisotropic properties into finite element (FE) models of the brain. This has also allowed the development of subject-specific FE models of the brain, which has proven to be useful in analyzing wide ranges of different brain responses after head impact, a hallmark of TBI.

### 4.2 Impact of cardiac ventricular measurement and models

Continuous interaction between cardiac walls and blood stimulates a change in wall architecture, with alterations in the anatomy of flow being suggested as the cause of remodelling. Building models of flow in paediatric hearts is essential to understand the development of early biomarkers and predictors of cardiovascular disease in a step towards improvement of cardiac disease outcome. Personalized biventricular models have paved the path to advanced and in-depth cardiac analysis. In addition to the routine cardiac indices that are now easier and more robust in the way they are measured; new predictive indices are gaining interest such as strain measurements (Elsayed et al., 2022). Ultimately, our research will lead to the development of paediatric cardiac atlases. Building atlas from cardiac models has taken computational cardiac modelling into a new era of quantification of variations in related age and pathology groups to help with disease prediction by scoring changes and associating them with risk factors (Mauger et al., 2019). Advanced biomechanics models have enabled shape scoring and quantitative analysis of regional cardiac shape changes with age and pathology progression. 4D flow acquisitions have added real time visualization and quantification of flow inside the ventricles and major vessels adding another layer of knowledge.

### 4.3 Why we need pulmonary system modelling

The conducting airways of the lung and their accompanying vasculature are all present at birth, but the alveolar airways progressively develop throughout childhood and into adolescence. Similarly, the chest wall, which plays a major role in patterns of breathing, becomes less compliant through early life leading to a transition between a young child predominantly “chest breathing” to a more adult-like pattern of “diaphragm breathing.” It is not clear at what age respiratory system development can be considered “complete.” However, it is clear that the compliance of the lung changes with growth right up to the late teens. Biophysical structure-based modelling of the lung, which seeks to explain lung function from its structure, has been dominated by the use of ionizing CT imaging to provide structural information. This is limited-to-impossible for imaging normal structure in children, or for follow-up imaging in many paediatric patients. Improvements to identification and treatment of chronic conditions such as bronchiectasis and cystic fibrosis means that life-expectancy with these conditions is increasing, and radiation exposure matters more than ever. Model guided analyses will provide a pathway for ensuring that multi-modal imaging obtained clinically can appropriately be used for diagnosis (i.e., using CT) and for longitudinal assessment of disease progression (i.e., using MRI), particularly in children.

Our MRI-based approach, when applied to a larger cohort, will enable us to build a first of its kind paediatric child-specific model of the thoracic cavity, airways and pulmonary vessels using a safe and repeatable imaging modality. The ability to predict ventilation and perfusion distribution adds an additional fidelity to the predictive power of such a model. Integration with the cardiac models described here will capture the whole cardio-respiratory function.

The potential long-term impact of this study on managing childhood respiratory disease are two-fold: 1) Many diagnostic criteria for lung disease consider children to be small adults, which they are not (Clark et al., 2011; Clark et al., 2019; Di Cicco et al., 2021). Accounting for anatomical and functional differences between paediatric and adult lungs will contribute to better understanding of the symptoms of childhood respiratory disease and how they evolve through childhood. Understanding developmental modifications to the respiratory tract will be helpful for management of chronic airway disease. 2) Repeated or severe childhood respiratory infections can influence lung development and life-long respiratory health (Oakes et al., 2018). Personalized models of the lung through childhood will provide a tool for better interpreting the change in medical imaging and diagnostic tests at follow-up timepoints.

### 4.4 Why we need bone measurement and models

Skeletal growth in children is characterized by progressive modelling of the skeleton in response to a complex interplay between biological and mechanical factors (Carter, 1987). As the limbs of the typically developing (TD) child grow, there is a programmed modelling of the long bones with systematic changes in length, width, and version (Herring and Tachdjian, 2020). Atypical development, such as children suffering from cerebral palsy or slipped capital femoral epiphysis (SCFE), can lead to delayed or altered skeletal modelling with consequent effects on hip stability, gait pattern and limb alignment (Gage et al., 2009; Hosseinzadeh et al., 2020). Understanding morphological variation in the typically developed skeleton is important to diagnose and treat morphological variations and pathology. It is well known that bone increases in size with age as a child grows until growth plate fusion occurs, which can be up to 20 years of age in the lower limb (Herring and Tachdjian, 2020). It is not well known whether gender and ethnicity affect linear and angular bone measurements. Our approach will help answer such questions.

### 4.5 Why we need skeletal muscle models

Lower limb muscle modeling is relevant to understanding gait and ambulation; this is an important functional aspect of growth and development in paediatric and adolescent populations. Whole body image sets can be used to generate muscle models for the upper limb and trunk, and to correlate these with measurements of growth for other organs such as the heart and lungs.

Skeletal muscles are the unique driver of motion for humans and animals. The healthy growth and development of skeletal muscles during childhood and adolescence is essential to a healthy adulthood. This is exemplary in the lower limb muscles, where the correct development and use of the muscles contributes to the proper development of the skeleton (Nowlan et al., 2010); in conditions such as cerebral palsy, impaired muscle mechanics contributes to bony deformities that often require surgery to resolve (Quinby et al., 2005; Graham et al., 2021). Beyond the mechanical contribution of muscles to skeletal development, and the importance of healthy musculature for healthy gait and overall mobility, skeletal muscle contributes considerably to other aspects of physiology, such as glucose uptake (Mizgier et al., 2014) and as a protein reservoir for metabolism (Ruderman, 1975; Sartori et al., 2021). Given its importance in growth and into adulthood, it is surprising that so little is known about the correct progression of skeletal muscle volume and morphology in childhood and adolescence. The authors are unaware of longitudinal data on muscle size and shape profiles in children and adolescents. Acquisition of such important data will allow for an understanding of the normal growth progression in humans, assessment of atypical growth, early diagnosis of abnormal growth conditions, and an opportunity for early intervention in these cases.

### 4.6 Roadmap

This work is the first step towards achieving the integration of imaging and modelling towards improving our understanding of the human body in health and disease and thereby help improve clinical diagnosis and treatment of a range of medical conditions. In this work, we have run multiple existing computational physiology workflows that perform a series of processing steps to personalize computational models using structural and functional measurements from individuals, e.g., imaging or waveform data. Computational modelling provides the unique advantage of improving our ability to interpret and understand large amounts of disparate data to elucidate structure-function relationships (Gorgolewski et al., 2016; Hunter, 2016). We have demonstrated in this study that we can apply our existing workflows to generate personalized models of organs from imaging data. The uniqueness of our study is that the data, and hence the generated personalized models, were acquired from the same child. Such a dataset has numerous benefits, including allowing structure-function relationships to be understood across different organ systems and overcoming existing challenges where data from only a single organ is available. In future, we intend to use the personalized models to perform cross-sectional analysis across a cohort of children. This will help establish normative ranges of structure and function in children and quantify their variability in a healthy population. Quantifying this variability will help identify robust baseline measures against which threshold values for diseases are defined in national/international guidelines.

Our approach also enables novel “next-generation” workflows to be developed including the linking of different organ systems, by leveraging and reusing the personalized models we have already created. For example, understanding the interplay between the musculoskeletal system and neurological control in cerebral palsy may help further improve therapies compared with considering the musculoskeletal alone. Furthermore, establishing normal ranges may be important for the early diagnosis of such diseases. To facilitate the reuse of data to enable such developments, we will store models, measurements, and associated workflow results in a standardized data structure [e.g., the Brain Imaging Dataset Structure (BIDS) (Bandrowski et al., 2021) or the SPARC Dataset Structure (SDS) (Wilkinson et al., 2016)] to meet fair principles (Cassilhas et al., 2016).

In this study, we showed how existing computational physiology workflows that were built for a specialized purpose could be used to create personalized models of each organs in a child. For example, the lung modelling workflow generated three-dimensional models all in the same consistent geometrical framework. In this manner, different workflows can be applied to the same dataset. We aim to integrate the outputs of each of these workflows in the future to create a virtual physiological human or “digital twin” of that individual. The creation of digital twins can help to improve the understanding, prevention, diagnosis and treatment while considering comorbidity influences. This initiative is being led by the Auckland Bioengineering Institute (ABI) as part of the International Physiome Project for the International Union of Physiological Sciences (IUPS). The ABI is also a founding member of the Virtual Physiological Human (VPH) institute, an international not-for-profit organization, whose mission is to ensure that the Virtual Physiological Human is fully realized, universally adopted, and effectively used both in research and clinic. This research will help contribute valuable data for delivering this vision and help towards establishing the first digital twin of children.

The anatomical and functional definition provided by the computational models here presented is essential to understand the proper function of the physiology of an individual. As different systems are highly integrated to each other, deficiencies in one system can result in severe effects in others. For example, there is a large body of evidence linking brain health with physical exercise—that is, which links the actuation of the musculoskeletal system with the positive benefits to the brain (Wu et al., 2018; Hu et al., 2020). At the same time, systemic conditions such as hypertension involves many systems, as it results from vascular adaptation mediated by the nervous system depending on the integration of different baro- and chemoreceptors deployed in the vasculature [aortic arch baroreceptors and carotid body chemoreceptors (Stillman et al., 2020)], brain [astrocytes end-feet bororeceptors (Boron and Boulpaep, 2016; Marina et al., 2020; Wu et al., 2020)] and lungs (stretch baroreceptors). Even homeostasis and allostasis processes usually rely on the interaction of these different systems. For example, a person running will have an active adaptation of the cardiovascular (systemic blood pressure and local blood flow regulation depending in part by cardiac output and frequency) and respiratory (gas exchange) systems, affected by muscular action that promotes venous return and constraints tissue perfusion resistance, and orchestrated by autonomous and central nervous systems. In all these cases, only an integrated physiology approach will yield a comprehensive and personalized assessment of the health status and progression of disease. Our study was partly motivated by a growing need to understand growth in children who cannot be assumed to be scaled down models of adults: not just in structure but also in function. Many areas of research unfortunately make this assumption. Our study will in part help understand early development through MRI and computational models.

## 5 Limitations

The proposed methodology has some practical limitations. First, it excluded the early childhood and post-puberty group as a matter of convenience and logistical ease. Hence the immediate benefits are mostly seen in the paediatric population of this narrow and specific age group. In other words, age-associated changes in physiology cannot be comprehensively studied across a wider age range. Second, the choice of organs and organ systems were not exhaustive. For example, many early biomarkers of neurological diseases may be detectable in the orbits, muscles, microvasculature, etc., of the eyes. But the current methodology did not include such models. Third, the technology to understand structure-functional relationships and organ-to-organ interactions are not fully mature in some research areas. Hence this may limit the ability of achieving fully functional workflows in the immediate future. Despite these limitations, given the growing interest in adopting data and model sharing standards, we speculate that the present work—which includes both advanced imaging and computational models—will create important avenues for collaboration and helping to fast track the development of inter-organ frameworks.

### 5.1 Role of MRI

While some disease conditions are routinely diagnosed by computed tomography, including in children, great care must be taken to ensure that radiation exposure in a child is not excessive over a lifetime. MRI is a safe alternative and new imaging protocols are being developed for clinical applications. For example, diagnosis of bronchiectasis and cystic fibrosis using MRI are gaining momentum. Similarly, imaging of bone has recently become possible with the Zero TE technique (Cho et al., 2019; Eley and Delso, 2021). With advances in imaging and reconstruction technologies and further emergence of deep learning-based segmentation, the scan times are becoming shorter without compromising image quality making it particularly well suited for children. In our study, it can be noticed that many sequences are only 1–3 min long. Hence developing imaging protocols and accompanying image analysis methods using MRI as the modality of choice holds great promise for the future.

## Data Availability

The datasets presented in this article are not readily available because Mātai medical research institute are gaurdians of this imaging data. Any requests for sharing imaging data will be taken on a case-by-case basis in consultation with the local advisory board. Requests to access the datasets should be directed to l.potter@matai.org.nz.
